# An overview of public health education in South Asia: Challenges and opportunities

**DOI:** 10.3389/fpubh.2022.909474

**Published:** 2022-08-26

**Authors:** Chandanadur Thippaiah Anitha, Konok Akter, Kalyankar Mahadev

**Affiliations:** ^1^School of Medical Sciences, University of Hyderabad, Hyderabad, India; ^2^Health Systems and Population Studies Division, International Centre for Diarrhoeal Disease Research, Dhaka, Bangladesh

**Keywords:** career opportunities, challenges of public health education (PHE), higher education in PH, MPH program, South Asia

## Abstract

Over the past two decades, there has been an increased demand for Public Health Education (PHE) in South Asia. While this region has a large number of Public Health (PH) institutions, the quality of PHE has not been aligned with the core PH competencies. In this article, we present an overview of Master of Public Health (MPH) programs across South Asian countries. An extensive systematic search on various web search engines regarding PH course offerings was conducted, including specific institute and educational websites. By 2021, more than 180 institutions in South Asia provided an MPH degree. Most of these institutions/universities were found in India, Pakistan, and Bangladesh, and a few among these institutions were established as independent Schools of Public Health (SPH), separate from medical colleges, and had a multidisciplinary faculty. But, dedicated training facilities in the specialized field of public health were not found in most of these institutions. Generally, a well-defined MPH curriculum is not currently available except in India where the University Grants Commission (UGC) guideline for a model MPH curriculum has been proposed by the Ministry of Health and Family Welfare. The entry criteria for an MPH degree in India is accepting students in multidisciplinary fields, while in other South Asian countries this is primarily restricted to medical/paramedical students with a basic understanding of preventive medicine. The aim of this review was to document the current and future PHE opportunities and challenges in South Asia.

## Introduction

Public health (PH), an interdisciplinary field takes into account not only physical ailments but also incorporates psychological and social well-being. PH encompasses the science and art of preventing diseases, prolonging life, and improving quality of life through organized efforts and informed choices of society, organizations, communities, and individuals (1). In 1978, the Alma Ata Conference restated the critical role of PH in accomplishing health for all by addressing the importance of equity, community participation, and inter-sectoral collaboration (2). The determinants of health in the population and analysis of the threats are grounded in the PH approach (3).

For the growth and prosperity of a nation, health is a key aspect to be considered (4). The field of PH encompasses various courses including epidemiology, bio-statistics, management of health services, environmental health, community health, behavioral health, health economics, public health policy, health politics, occupational safety including sub-fields such as disability, gender issues in health, mental health, and maternal and child health (5). On an international level, conflicting viewpoints on the approach to public health issues may arise, for instance, the goal of preventive vs. curative services, selective vs. comprehensive primary health care, or integrated (horizontal) vs. top down (vertical) programs (6). Intervention strategies that incorporate the PH sector's collaboration must contain the establishment of appropriate graduate and post-graduate study opportunities in PH.

## Significance of the review

The South Asian countries form the South Asian Association for Regional Cooperation (SAARC) including Afghanistan, Bangladesh, Bhutan, India, Maldives, Nepal, Pakistan, and Sri Lanka. The SAARC nations are home to nearly one-fifth of the world's population and have been suffering from a vast number of health-related challenges such as a double burden of infectious and non-communicable diseases (NCDs), malnutrition, unsafe pregnancies, and a rapidly escalating plethora of NCD epidemics (7). Even though PH existed for over a century, it is still an evolving field in SAARC nations. PH knowledge is necessary to help students develop community health by organizing, evaluating, and implementing effective and equity-based PH programs (8). However, the multidisciplinary field of PH needs coordinated efforts with teams of PH workforce, specialists, and professionals from diverse sectors who contribute to the broader field of PH (9). The SPH is better placed to offer comprehensive health professional education, conduct multidisciplinary research, population studies, and adopt collaborative partnerships to achieve the sustainable development goals (SDGs) (9). With an aim to examine the challenges and opportunities in professional PHE in South Asia, a brief review was undertaken.

## Review of the literature

MPH programs in South Asia were explored by Google scholar, CINAHL, Pub Med, Web of Science, ELDIS using keywords-PHE, South Asia, SAARC nations, higher education, study programs, MPH/M.Sc., career opportunities for PH graduates, challenges of PHE in SAARC nations. The Masters of Public Health (MPH) program is of 2 years duration as per the University Grants Commission standards (10). Post Graduate Diploma in Health Science, which is comparable to a 1-year MPH, was not included. The University websites, and education-based websites including Collegedunia.com (11), career360 (12), Eduvision (13), Collegedekho (14) were searched. Further, information on the SAARC nation's PHE, current situation, and challenges was obtained from the World Health Organization (WHO) and the Centre for Disease Control and Prevention (CDC) country pages. Articles in other regional languages apart from English, Bachelor's degree in Public Health, and doctoral programs were excluded.

## Current status of public health education in SAARC nations

### India

In India, 95 institutions were identified that provide MPH programs of 2 years duration with English as the medium of instruction. The number of MPH programs has increased dramatically during the last two decades and specifically over the last couple of years with the onset of the COVID-19 pandemic (15). However, it is necessary to recognize that the curricula and criteria for admissions in these programs are diverse (16). The universities follow the Model Curriculum floated by the Ministry of Health and Family Welfare (17). A new National Education Policy (NEP 2020), replacing the National Policy on Education from 1986, addressing the importance of research and a multi-disciplinary approach has been approved by the Union Cabinet of India (18). Under the NEP 2020, Higher Education Institutes (HEIs) in India have a choice of providing a 1 year Master's degree for those who have done their Bachelors degree of 4 years that includes research component in the programme (19). A 2-year Master's program with research activities in the second year is offered for those who obtained a bachelor's degree in 3 years (18). The NEP 2020 allows students to experience research-based learning on par with the academic structure of HEIs in developed countries (18, 19).

The MPH program includes five traditional core areas proposed by the Association of Schools and Program of Public Health (ASPPH). These disciplines are Epidemiology, Bio-statistics, Environmental Health Sciences, Health Policy and Management, Social and Behavioral Sciences. Some universities offer unique non-core subjects such as global health technology in PH by Kalinga Institute of Industrial Technology, food and nutrition courses and field posting at Career Point University. Medical colleges have traditionally been the primary source of PH specialty training in India (20). Outside of medical institutions, there has been an intentional shift in the past decade toward the establishment of SPH that allow non-medical personnel to obtain academic competencies in PH (21). Incidentally, India is the largest educational hub among the SAARC nations and accepts students from neighboring and other foreign nations to enroll in HEIs offering an MPH course. The Government of India through the Indian Council for Cultural Relations (ICCR) offers scholarships for students from SAARC nations and the MPH course has gained increased demand over the past 2 years. The private-public partnership (PPP) model too has boosted MPH programs that provide PHE to students in the region. The Public Health Foundation of India (PHFI) remains a one-of-a-kind private–public collaboration that aims to reinvigorate PH by pooling resources from the government and private philanthropy to address the most pressing public health issues (22).

### Bangladesh

In Bangladesh, 32 institutions offer MPH programs as of mid-year 2022. At Jahangirnagar University, there has been a M.Sc. in PH degree program for which only B.Sc. in PH students of this department can enroll. Most of the courses are of 1-year duration and the medium of instruction is English, while some courses are 16/18 months in duration. There is only one University (First capital) that provides an MPH degree with 2-year duration for students from a non-medical background. The most reputable University for an MPH degree is the James P. Grant School of Public Health established in 2005 at BRAC University. This program offers an innovative 12-month MPH curriculum that begins with 6 months of training in basic PH skills in the context of rural health action on the Savar rural campus, followed by training on the BRAC University campus (23).

### Pakistan

In Pakistan, PH programs are available in 44 institutions including the MPH and MSPH degrees. Most of the courses were regular (2 year duration) and full time. The Provincial Health Services Academy provides a 4-year program and the Institute of PH and Gandhar University offer a 1-year MPH program. The entry criteria include medical/non-medical background and few universities require work experience to enroll in the MPH program (e.g., Jinnah Sindh Medical University). Very few universities provide training to graduates in primary health care and research including Jinnah Sindh Medical University.

### Nepal

Ten universities provide MPH programs of 2 years duration, except Manmohan Technical University and Tribhuvan University, which offer programs with a duration of one and half years and 1 year, respectively. Some universities ask for the TOEFL/IELTS test as a pre-requisite for admission (notably Purbanchal University in Biratnagar).

### Bhutan, Maldives, Afghanistan, and Sri Lanka

In Bhutan, Bachelor's of Public Health is offered by Khesar Gyalpo University of Medical Sciences (KGUMSB). There is no MPH program offered.

The Maldives higher education system consists of four universities with a total of 40 study programs. Two universities, the Maldives National University and the Villa College University, provide MPH program with English as the medium of instruction. However, there is no core course offered in Statistics.

In Afghanistan, a total of nine provinces have 87 universities. The website of universities was not updated and meager information was available. Only two universities appeared to offer an MPH credential with the medium of instruction as English. The Minister of Higher Education in partnership with WHO formally created the MPH program at Kabul Medical University (KMU) in the year 2013. Another MPH program was developed at Kandahar University with funding from the United States of America Agency for International Development (USAID), University Support and Workforce Development Program (USWDP), and technical help from Johns Hopkins University in the United States of America (23).

In Sri Lanka, the University Grants Commission provides oversight of 17 Sri Lankan universities and other educational institutions, all of which are classified as HEIs. A joint MPH program is provided by two institutions: the University of Kelaniya and the University of Peradeniya. Thus, by 2022 nearly 183 institutions in SAARC nations provided an MPH degree (Table 1).

**Table 1:** List of institutions offering public health education in SAARC nations.

**India**
1. Achutha Menon Centre for Health Science Studies, Sree Chitra Tirunal Institute for Medical Sciences and Technology, Thiruvananthapuram, Kerala
2. Adesh University, Bathinda, Punjab
3. Akal School of Public Health, Eternal University, Sirmour, Himachal Pradesh
4. All India Institute of Hygiene and Public Health, Kolkata, West Bengal
5. All India Institute of Medical Sciences, Jodhpur, Rajasthan
6. All India Institute of Medical Sciences, Rishikesh, Uttarakhand
7. All India Institute of Medical Sciences, Raipur, Chhattisgarh
8. Amity University, Noida, Uttar Pradesh
9. Amrita Institute of Medical Sciences and Research Centre, Kochi, Kerala
10. Asian Institute of Public Health, Bhubaneswar, Odisha
11. Athar Institute of Health and Management Studies, Gautam Nagar, New Delhi
12. B. J Government Medical College, Pune, Maharashtra
13. Career Point University, Kota, Rajasthan
14. Central University of Kerala, Kasargod, Kerala
15. Central University of Tamil Nadu, Thiruvarur, Tamil Nadu
16. Centre for Emerging Areas in Science and Technology, Punjab University, Chandigarh
17. Chitkara University, Chandigarh, Punjab
18. Christian Medical College, Vellore, Tamil Nadu
19. Datta Meghe Institute of Medical Sciences, Wardha, Maharashtra
20. Delhi Pharmaceutical Sciences and Research University, Pusp Vihar, Delhi
21. Dr. Shankarrao Chavan Government Medical College, Nanded, Maharashtra
22. Dr. Rammanohar Lohia Avadh University, Ayadhya, Uttar Pradesh
23. Edward and Cynthia Institute of Public Health, Mangaluru, Karnataka
24. Eternal University, Sirmour, Himachal Pradesh
25. Ganpat University, Kherva, Gujrat
26. GD Goenka University, Gurgoan, Haryana
27. Global Institute of Healthcare Management, Najafgarh, Delhi
28. Global Institute of Public Health, Thiruvananthapuram, Kerala
29. Government Medical College, Akola, Maharashtra
30. Government Medical College, Aurangabad, Maharashtra
31. Government Medical College, Chandrapur, Maharashtra
32. Government Medical College, Gondia, Maharashtra
33. Government Medical College, Nagpur, Maharashtra
34. Grant Government Medical College, Mumbai, Maharashtra
35. Guru Gobind Singh Indraprastha University, New Delhi
36. ICMR- National Institute of Epidemiology, Chennai, Tamil Nadu
37. I K Gujral Punjab Technical University, Khapurthala, Punjab
38. Institute of Clinical Research India (ICRI), Sam Global University, Bhopal, Madhya Pradesh
39. Indian Institute of Health Management Research (IIHMR) Jaipur, Rajasthan
40. Indian Institute of Public Health - Delhi
41. Indian Institute of Public Health - Gandhinagar, Gujarat
42. Indian Institute of Public Health - Hyderabad, Telangana
43. Indian Institute of Public Health – Shillong, Meghalaya
44. Institute of Management studies, Kolkata, West Bengal
45. Institute of Public Health, Kalyani, West Bengal
46. Interdisciplinary School of Health Sciences, Savitribai Phule Pune University, Maharashtra
47. Jagadguru Sri Shivarathreeswara University, Mysuru, Kamataka
48. Jawaharlal Institute of Postgraduate Medical Education and Research, Puducherry
49. Jawaharlal Nehru University, Munirka, Delhi
50. Jodhpur School of Public Health, Jodhpur, Rajasthan
51. Karnatak Lingayat Education University, Belgaum, Karnataka
52. Karnataka State Rural Development & Panchayat Raj University, Gadag, Karnataka
53. Kalinga Institute of Industrial Technology (KIIT), Bhubaneswar, Odhisa
54. KPC Medical College, Kolkata, West Bengal
55. Krishna Institute of Medical Sciences, Karad, Maharashtra
56. Maharashtra University of Health Sciences, Nashik, Maharashtra
57. Mahatma Gandhi University, Kottayam, Kerala
58. Mahatma Jyoti Rao Phoole University, Jaipur, Rajasthan
59. MD Goenka University, Sohna, Haryana
60. MIT world peace University, Pune, Maharashtra
61. M.S. Ramaiah University of Applied Sciences (MSRUAS), Bengaluru
62. National Centre for Disease Control, Sham Nath Marg, Delhi
63. National Institute of Mental Health and Neuro Sciences, Bengaluru, Karnataka
64. NITTE University, K.S. Hegde Medical Academy ( KSHMA) Mangaluru, Karnataka
65. NSHM Knowledge Campus (NSHMKC), Kolkata
66. Noida International University, Gautam Budh Nagar, Uttar Pradesh
67. Om sterling global University, Hisar, Haryana
68. Padmashree School of Public Health, Bengaluru, Karnataka
69. Punjab University, Punjab
70. Parul University, Ahmedabad, Gujarat
71. Post Graduate Institute of Medical Education and Research, Chandigarh, Punjab
72. P P Savani University, Surat, Gujarat
73. Prasanna School of Public Health, Manipal University, Manipal, Karnataka
74. Pravara Institute of Medical Sciences, Ahmednagar, Maharashtra
75. Rabindranath Tagore University (RNTU), Bhopal, Madhya Pradesh
76. Rajiv Gandhi Institute of Public Health and Centre for Disease Control, Karnataka
77. Rayat Bahra University, Mohali, Punjab
78. Shalom Institute of Health & Allied Sciences, SHUATS, Allahabad, Uttar Pradesh
79. Sri Devaraj Urs Academy of Higher Education and Research, Kolar, Karnataka
80. Sai Group of Institutions (SGI)‘, Dehradun, Uttarakhand
81. Sam Higginbottom Institute of Agriculture, Technology and Sciences, Uttar Pradesh
82. Sri Ramaswamy Memorial Institute of Science and Technology, Chennai, Tamil Nadu
83. SGT University- Faculty of Engineering and Technology (SGTU), Gurugram, Haryana
84. Shri Ramasamy Memorial (SRM) Institute of Science and Technology, Chennai, Tamil Nadu and Gangtok, Sikkim
85. Sri Ramachandra Medical College and Research Institute, Chennai, Tamil Nadu
86. Symbiosis Institute of Health Sciences, Pune, Maharashtra
87. Tata Institute of Social Sciences, Mumbai, Maharashtra
88. The Global Open University, Dimapur, Nagaland
89. The Tamil Nadu Dr.M.G.R Medical University, Chennai, Tamil Nadu
90. University of Hyderabad, Hyderabad, Telangana
91. University of Lucknow, Lucknow, Uttar Pradesh
92. Utkal University, Bhubaneswar, Odisha
93. University of Technology Sanganer, Jaipur, Rajasthan
94. Vinayaka Mission's Research Foundation, Salem, Tamil Nadu
95. Vinayaka Mission's Research Foundation - School of Allied Health Sciences (AVIT), Puducherry
96. Yenepoya Medical College, Mangaluru, Karnataka
**Bangladesh**
1 American International University-Bangladesh, Kuratoli, Dhaka
2 ASA University Bangladesh, Dhaka
3 Atish Dipankar University of Science and Technology, Dhaka
4 Bangabandhu Sheikh Mujib Medical University, Dhaka
5 Bangladesh University of Health Sciences, Dhaka
6 Bangladesh University of Professionals, Dhaka
7 BRAC University, Dhaka
8 Fareast University, Dhaka
9 First Capital University of Bangladesh, Chuadanga
10 Hamdard University Bangladesh, Gazaria, Dhaka
11 Independent University Bangladesh, Dhaka
12 Islamic University Bangladesh, Kushtia
13 Jagannath University, Dhaka
14 Jahangirnagar University, Dhaka
15 Leading University Bangladesh, Sylhet
16 National Institute of Preventive and Social Medicine (NIPSOM), Dhaka
17 North East University Bangladesh, Dhaka
18 North South University, Dhaka
19 North Western University, Khulna
20 Northern University, Bangladesh, Dhaka
21 Bangladesh Open University, Dhaka
22 Premier University, Chittagong
23 Pundra University of Science & Technology, Rangpur
24 Rajshahi University, Rajhshahi
25 Ranada Prasad Shaha University, Narayanganj, Dhaka
26 State University of Bangladesh, Dhaka
27 United International University, Dhaka
28 University of Comilla, Comilla
29 University of Creative Technology, Chittagong, (UCTC)
30 University of South Asia, Dhaka
31 Varendra University, Rajhshai
32 Z H Sikder University of Science & Technology, Shariatpur
**Pakistan**
1 Afro-asian Institute, Lahore
2 Al- Hamd Islamic University, Quetta
3 Allied College of Health Sciences, Khanewal
4 Allied College of Health Sciences, Multan
5 Armed Forces Postgraduate Medical Institute, Rawalpindi
6 Baqai Medical University/hospital, Karachi
7 Buraq Institute of Higher Studies, Peshawar
8 Dow University of Health Sciences, Karachi
9 Federal Institute of Health Sciences, Lahore
10 Federal Institute of Health Sciences, Multan
11 Fedral Institute of Health Sciences, Muzaffarabad
12 Frontier Institute of Medical Sciences, Abbottabad
13 Gandhara University, Peshawar
14 Gomal University, D.i. Khan
15 Institute of Computer and Management Sciences(icms), Peshawar
16 Institute of Health & Management Sciences, Islamabad
17 Institute of Health Sciences, Mardan
18 Institute of Public Health, Lahore
19 Institute of Public Health, Quetta
20 Islamabad Federal College F-10, Islamabad
21 Khyber Medical University, Peshawar
22 Liaquat University of Medical and Health Sciences, Jamshoro
23 Mardan Institute of Sciences, Mardan
24 National University f Medical Sciences, Rawalpindi
25 Pakistan Institute of Community Ophthalmology, Peshawar
26 Peoples University of Medical and Health Sciences for Women, Nawab Shah
27 Peshawar Institute of Modern Sciences, Peshawar
28 Peshawarcity Institute of Modern Sciences, Peshawar
29 Prime Institute of Public Health, Peshawar
30 Provincial Health Services Academy, Peshawar
31 Shaheed Mohtarma Benazir Bhutto Medical University, Larkana
32 The Next College, Multan
33 The University of Lahore (Main Campus), Lahore
34 Times Institute, Multan
35 Udhyana Instititue Of Medical Sciences, Abbottabad
36 University of Health Sciences, Lahore
37 Vertex College of Science and Technology, Islamabad
**Nepal**
1 B.P. Koirala Institute of Health Sciences, Dharan
2 Central Department of Public Health, IOM, Kathmundu
3 Chitwan Medical College, Chitwan
4 Kathmandu University School of Medical Sciences, Kathmundu
5 ManMohan Memorial Institute of Health Sciences, Nagarjun
6 Nobel College, Kathmandu
7 Om Health Campus, Purbanchal University
8 Patan Academy of Health Sciences, Lalitpur
9 Pokhara University, Pokhara
10 Purbanchal University College of Medical and Allied Sciences, Biratnagar
11 Tribhuvan University, Kathmundu
**Countries** **Institutions**
**Bhutan, Maldives, Afghanistan, Sri Lanka**	
Bhutan Khesar Gyalpo University of Medical Sciences (KGUMSB), Thimpu*, *No Master degree offer in PH
Maldives 1. Maldives National University, Male 2. Villa College universities, Male
Afghanistan 1. Kabul Medical University (KMU), Kabul 2. Kandahar University, Kandahar
Sri Lanka 1. University of Kelaniya, Kelaniya 2. University of Peradeniya, Peradeniya

## Core competencies of public health education

The essential minimum set of attributes, such as applied knowledge, skills, and attitudes, that enable an individual to perform a set of tasks to an appropriate standard efficiently and effectively is defined as core competencies (24). Core competencies provide a common shared language for all PH professions to define what all are expected to be able to do to work optimally (25).

**Figure 1:**
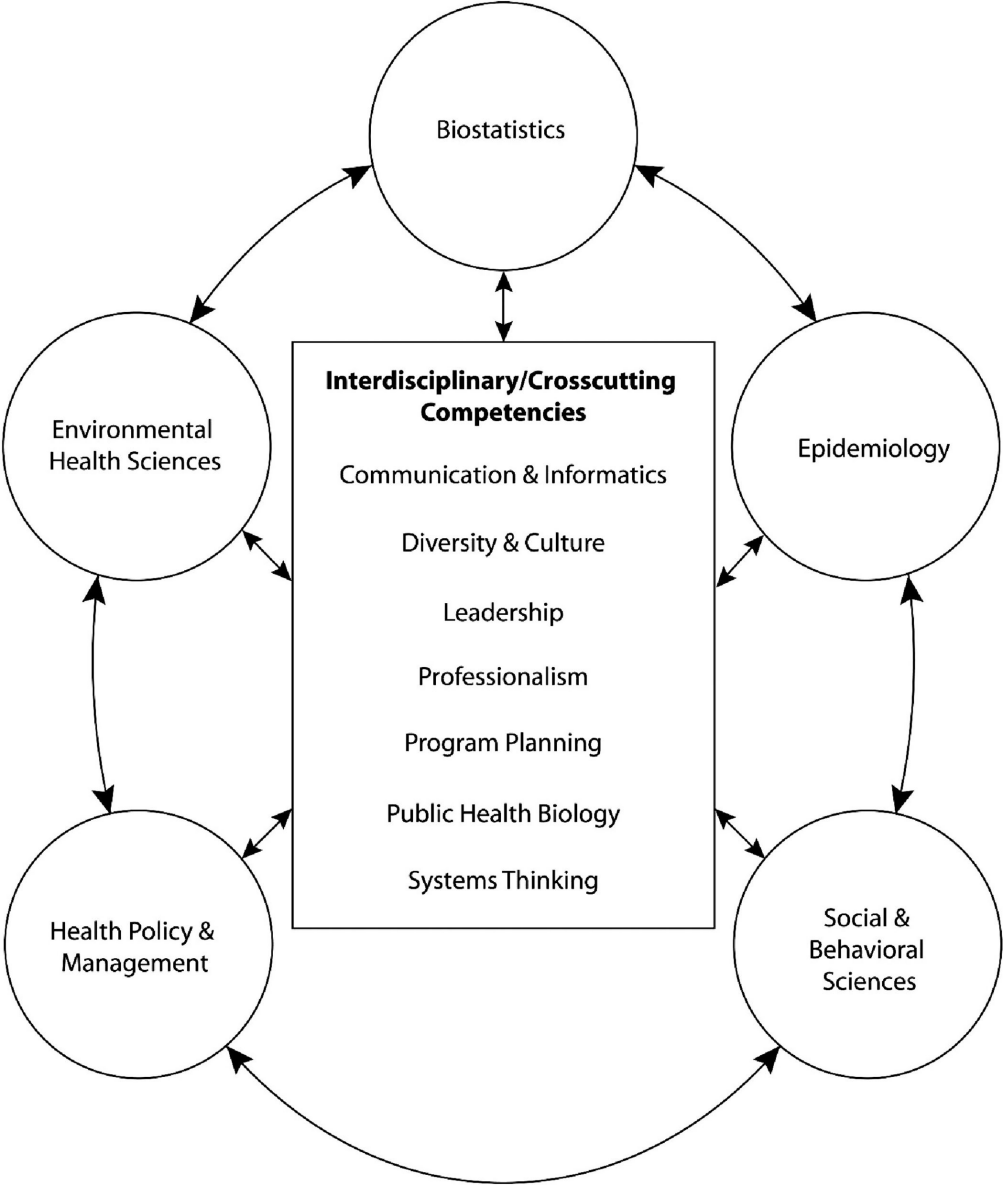
Core competency model for the MPH degrees proposed by Association of Schools and Program of Public Health (ASPPH) 30.

The COVID-19 pandemic has underlined the necessity of core competencies in performing PH functions such as disease outbreak prevention, detection, and response (26). However, most resource-poor countries struggle to impart the necessary PH competencies to the public health personnel to conduct these and other PH duties successfully (22, 27). In order to effectively deliver the critical PH functions such as epidemiological surveillance, situation assessments, and health promotion required that the PH professional should have fundamental competencies (28, 29). The functions are multidisciplinary in nature and not limited to a single program or topic, and every staff at all levels in the PH system should have these core skills established (26). Toward this goal, the core competencies of the Association of Schools and Program of Public Health (ASPPH) in the United States established by a national consensus in 2006 remain a useful resource and reference for PH educators, administrators, and students (30). The ASPPH proposed Core Competence in Public Health model includes competencies in five traditional PH core areas as well as seven interdisciplinary/cross-cutting areas (Figure 1). The MPH students graduated from a Council on Education for Public Health (CEPH)-accredited school or program of PH in the United States are equipped with these core competencies. The discipline specific competencies are Bio-statistics, Environmental Health Sciences, Epidemiology, Health Policy and Management, Social and Behavioral Sciences. The Interdisciplinary/Cross-cutting competencies include Communication and Informatics, Diversity and Culture, Leadership, Public Health Biology, Professionalism, Program Planning, Systems Thinking. The core competency assessment for MPH graduates in low and middle-income countries (LMICs) is uncommon and there exists a need for a Accreditation Council on PHE in the region (26, 30).

The WHO-ASPHER (Association of Schools of Public Health in the European Region) Competency Framework can be used as a starting point for more context-specific competencies to be developed (31). It contains three core categories: Content and context, Relations and interactions, and Performance and achievement.

Among South Asian countries, we found few studies regarding core competencies and one study emphasized the cross-cutting domain that included health communication and informatics, health management and leadership, professionalism, systems thinking, and PH biology along with the five core areas (32, 33). A study from Uttar Pradesh in India has identified core competencies for practicing PH professionals using a Delphi exercise (26). This study proposed 48 competency statements across eight domains: PH sciences, assessment and analysis, policy and program management, financial management and budgeting, partnerships and collaboration, social and cultural determinants, communication, and leadership.

## Challenges and opportunities of PHE for SAARC nations

The South Asian countries have similar health profiles to their neighboring countries. India, as the biggest among the SAARC nations in the region, has encountered challenges such as a scarce PH workforce; a lack of skilled workers in accordance with the health system needs of the population, and an unequal range of types and levels of ability of health workforce (34). There are gaps in PHE requiring in-depth understanding and addressing including the quantity and quality of PH education (35). The shortages in the health workforce are not the only issues, the limited availability of jobs, and lack of opportunities to absorb the recent MPH graduates within the health system, NGOs, and the industry seems a huge concern and this scenario was affecting the enrolment in higher studies in PH (36). In the wake of the pandemic, “Health” has become a central issue in our lives; thus, to attain it, the SAARC nations need to act in an organized manner. The curricula require to evolve in such a way that all levels of students can find the opportunities to enter the PH program and acquire training on health and human ecology (37). As PH deals with the health of the population as a whole, the term “Health literacy” can be a goal of our learning system in a true sense (38). Explicitly, the spread of health challenges and duties in society creates a demand for health training in a variety of vocations that do not include the word “health” in the title (37). A small percentage of students join MPH program by choice after undergraduate studies, prompted by the desire to work in the health-care field (22). However, research capabilities, financial accessibility, and innovation are strengths of SPH in India. But there is a need to improve collaborations and synchronize training with well-defined career routes (39).

Public health allied practitioners have always been viewed as being at the bottom of the health-care hierarchy (40). When there will be appropriate course curricula with evidence-based research opportunities and training in epidemiological studies, a better workforce in health care will be added. While there has always been a mismatch between supply and demand for healthcare workers in Bangladesh's healthcare sector, the situation has been different for allied PH practitioners and medical professionals (40).

Competence is critical in determining a health worker's capacity and preparedness for offering high-quality care (41). According to the findings of a study, academic institutions are creating PH graduates that lack the necessary abilities to operate in a variety of PH disciplines, and also the present MPH curriculum varies greatly between institutes with different emphasis (42, 43). A recent scoping analysis sheds light on the state of the MPH program across South Asian countries. Due to the lack of a comprehensive and consistent curriculum, the competencies acquired by these graduates may vary (44). WHO South-East Asia region (45) showed vast variation in institutes and courses offered regardless of the existence of numerous postgraduate courses and undergraduate courses in these countries. It demonstrates that there is a significant gap in our understanding of how effectively research outputs affect PH decision-making as a result of this gap between PHE and real-world PH policies and practices (46).

MPH is largely a professional practice degree in Nepal, as it is in most other SAARC nations. Despite this, MPH graduates are likely to work in research and academic settings due to the scarcity of possibilities for further education in PH (47). “Community Medicine” is an integral part of Nepalese medical school curricula. It was observed that a synergistic relationship between departments of PH and clinical sciences was not well integrated in actual practice (47). The notion that research has to be “community-based” has further discouraged interdisciplinary research within these institutions. The SPH in Nepal is located in educational institutions affiliated with specialized health centers, hospitals, and peripheral health facilities, all of which are engaged in the treatment and prevention of disease. Some of these diseases/conditions include infectious and chronic diseases, trauma, maternal and child health problems, and mental illness (47).

The literature available regarding PHE and training programs in Pakistan is scant. One study involving In-depth interviews with stakeholders revealed PH practitioners preferred to recruit someone with a medical degree (48). They further seek practical experience, skills in program coordination, resource mobilization, management, analytical skills, grant-writing abilities, strategic thinking, monitoring, and program evaluation as well as specific knowledge of Pakistan's health system. Investing in a PH training program is difficult as the government of Pakistan funds only 5% of the Health Service Academy's budget and the rest is to be raised through grants for capacity building and research (48). The literature on medical education has continued evidence of dispute on whether or not to include PH in a basic medical curriculum. Woodward, argues that the “clinical imperative of diagnosis and treatment is so firmly entrenched in the minds of students and in the cultures of medical schools that PH will always be diminished and elbowed to one side in medical curricula” (49). Usually, the dissimilarity is in the fundamental, philosophical, and practical differences between these two fields (50). The emphasis of PH is on the health of populations and stress prevention and health promotion, whereas clinical medicine focuses on the individual's health by concentrating on curative measures e.g., diagnosis and treatment (50). However, the COVID-19 pandemic highlighted the need for interdisciplinary collaboration to address the worsening pre-existing disparities and inequity that exacerbated the lack of access to health care around the world (51). The epidemic has raised awareness of the urgency for more funding for SPH and PH programs to create and execute new courses and techniques for acquiring key competencies (51, 52).

During the eighteenth and nineteenth centuries, the focus shifted to the development of personal preventive services and also the establishment of maternal and child health services including mass vaccination (53). To ensure “Health for all”, preventive practice in PH in parallel with the clinical practice was emphasized. By the early twentieth century, it was clear that PH was required to combat chronic diseases. Over the recent decades, it has become obvious that PH is needed to address the social, political, and commercial determinants of health, including through actions outside of the health system, such as in the agriculture sector, rural development, urban planning, and Health in all policies (53). Some studies document the surge in enrolment of MPH education following the onset of the COVID-19 pandemic. The applications to Masters in PH programs in the USA had seen a sharp increase (20%) for the academic year 2020-21 and were nearly 40,000, according to the Association of Schools and Programs of PH (54). Likewise, the number of institutions and universities offering MPH courses has gone up in South Asia specifically in India. But, there are no studies available in current literature that estimated the number of students successfully graduating with MPH degrees annually.

For obvious reasons, the COVID-19 pandemic has had a particularly important influence on the need for online education in PH. The MOHFW, Government of India has recognized these training programs as an innovative model of education in their National innovation summits (55). The holistic education envisaged under the NEP 2020 offers students at HEIs an internship with local industries/craftspeople as well as research internships at their own/other institutions. This enables them to actively engage with practical learning and, as a result, improve their skills and employability. The credits earned at foreign universities will be allowed to be counted toward the award of a degree if suitable and in accordance with the rules of each HEI (18). In Bangladesh, the government has big plans for digital and e-learning with the goal of making it a permanent part of the educational system (56). In Pakistan, The Prime Institute of Public Health offers competency-based PH courses that are practice-oriented to individuals with at least 14 years of education and interest in PH (57).

## Evaluation method of MPH program in South Asia

As discussed earlier, the content and structure of the WHO-ASPHER competency have proposed the development of a new approach to evaluating the MPH level programs, which highlighted that there is a wide range of competencies required to perform professional duties in PH (58). The choice-based credit system (CBCS) gives students the option of three different course types: Core, Elective, and Foundation. The CBCS is followed by most South Asian universities. But few universities lack the option of fundamental courses. Regarding the evaluation, we found that the method is mostly formative (Internally by the concerned faculty member through quizzes, tutorials, lab works, home assignments, class tests, class participation, term papers, and internal exams) and summative (Externally by the Office of the Controller of Examinations of concern University through year/semester-end examinations). In Bangladesh, private universities mostly follow the semester system, while public universities offer annual system courses; especially, while for other countries, majority of the universities offered semester system courses. The total credits varied across South Asian universities for MPH programs with most universities requiring more than 50 credits except Afghanistan (46 credit). The universities offered internship opportunities and facilitated the process, but a program-based research opportunities were generally lacking. Despite the vast scope, there is a strong necessity to improve cooperation and align training and employment paths as well as effective community engagement techniques in the region. An MPH program evaluation survey revealed that MPH graduates should be able to monitor health problems and epidemics in their communities, develop indicators and instruments to monitor and evaluate community health programs, develop proposals, apply biostatistics principles in public health, conduct operations/action research, understand social and community influences on public health, and involve the community in planning, delegating, and evaluating community health programs (33). In this review, the scarcity of literature on the evaluation of MPH programs in South Asia presents a challenge. A study on the transdisciplinarity of India's master's level public health programs revealed the lack of inclusivity of non-medical disciplines (59).

## Strengths and limitations of the review

This review provides an up-to-date perspective on the availability of rapidly emerging professional public health training and education programs in South Asia. The introduction of the National Education Policy, 2020 in India has large positive implications for Public Health Education nationally, regionally, and globally. This review updates the public health educational institutions that emerged in response to the pandemic in the region and generate greater awareness among the general public and PH professionals. Further, it emphasizes the need for a robust public health curriculum and core competencies for aspiring professionals who would constitute the future public health workforce in the region. However, there are some limitations, such as a lack of information pertaining to country-wise number of students' enrollment, professional accreditation, and regulatory bodies in South Asia to evaluate public health education.

## Conclusion

The professional PHE is evolving rapidly in the South Asia region. Among the eight SAARC nations, India has structured and well-defined PH course curricula. Indian universities and HEIs welcome foreign nationals for higher studies in PH. The literature and information on PHE are more readily available for India than for other SAARC nations. Till now Bhutan, Maldives, Afghanistan, and Sri Lanka have not expanded the scope of PHE in independent schools or separated it from medical schools. Some countries are strict in the eligibility criteria in Master's programs to only medical students (Sri Lanka, Afghanistan). Therefore, as PH is a multidisciplinary field, students from varied backgrounds should find the chance to enroll in the program. Since PH literacy is required for the recognition and fundamental grasp of how the social and physical environment influences health, it is a legitimate and worthwhile societal aim.

The emphasis on core competencies will be meaningful if it culminates with a job or higher education opportunities. To this effect, the engagement and involvement of academia, industry, and government stakeholders are essential. However, currently, there are no clear avenues for absorbing MPH graduates within the existing government health care infrastructure, NGO, or industries. A robust PH culture can be promoted by ensuring training facilities for students from both medical and non-medical backgrounds, a necessity in the twenty-first century. As the new PH graduates enter the government or development sectors, more opportunities to enroll non- medical graduates in the PH workforce become necessary. There is a lack of literature and critical analysis regarding PHE in the South Asia region. There is a compelling need to address the gaps and the consequent scope of PHE and job opportunities in the South Asian region. Given the spread of Institutes and Schools of Public Health in South Asia in recent decades, it would be worthwhile to evaluate the PHE in the region. South Asian countries must examine their human resource needs for health and create the capacity to meet education and health service needs in the future.

## Author contributions

CTA contributed to the conceptualization and project administration. CTA and KA contributed to the methodology. KA conducted the investigation and contributed to writing original draft. CTA and KM performed supervision and acquired resources, performed validation, contributed to writing, reviewing, and editing of the manuscript. All authors read and approved the final version of the manuscript for publication and their respective contributions.

## Conflict of interest

The authors declare that the research was conducted in the absence of any commercial or financial relationships that could be construed as a potential conflict of interest.

## Publisher's note

All claims expressed in this article are solely those of the authors and do not necessarily represent those of their affiliated organizations, or those of the publisher, the editors and the reviewers. Any product that may be evaluated in this article, or claim that may be made by its manufacturer, is not guaranteed or endorsed by the publisher.
